# Tetra-μ-benzoato-κ^4^ *O*:*O*′;κ^3^ *O*:*O*,*O*′;κ^3^ *O*,*O*′:*O*′-bis­[(benzoato-κ^2^ *O*,*O*′)(1,10-phenanthroline-κ^2^ *N*,*N*′)europium(III)] benzoic acid disolvate

**DOI:** 10.1107/S1600536810015229

**Published:** 2010-04-30

**Authors:** Ping Howe Ooi, Siang Guan Teoh, Chin Sing Yeap, Hoong-Kun Fun

**Affiliations:** a School of Chemical Sciences, Universiti Sains Malaysia, 11800 USM, Penang, Malaysia; b X-ray Crystallography Unit, School of Physics, Universiti Sains Malaysia, 11800 USM, Penang, Malaysia

## Abstract

The asymmetric unit of the title complex, [Eu_2_(C_7_H_5_O_2_)_6_(C_12_H_8_N_2_)_2_]·2C_6_H_5_COOH, contains one-half of the complex mol­ecule, the complete mol­ecule being generated by inversion symmetry, and one benzoic acid solvent mol­ecule. The two Eu^III^ ions are linked by four bridging benzoate ions, with an Eu⋯Eu distance of 3.96041 (12) Å. Each Eu^III^ ion is coordinated by one phenanthroline heterocycle and a bidentate benzoate ion. The irregular nine-coordinated geometry of the metal ion is composed of seven O and two N atoms. The mol­ecular structure is stabilized by intra­molecular C—H⋯O hydrogen bonds. In the crystal structure, mol­ecules are linked into chains by inter­molecular C—H⋯O hydrogen bonds along the *a* axis. The crystal structure is further stabilized by inter­molecular C—H⋯O and C—H⋯π inter­actions. Weak π–π inter­actions are also observed [centroid–centroid distances = 3.6962 (10)–3.6963 (10) Å].

## Related literature

For general background to and applications of europium(III) complexes, see: Yam & Lo (1999 ▶); Beeby *et al.* (2003 ▶); Tang *et al.* (2008 ▶); Faulkner *et al.* (2005 ▶). For related *Ln*–benzoato complexes (*Ln* = lanthanide), see: Niu *et al.* (1999 ▶, 2002 ▶); Shi *et al.* (2001 ▶); Ooi *et al.* (2010 ▶).
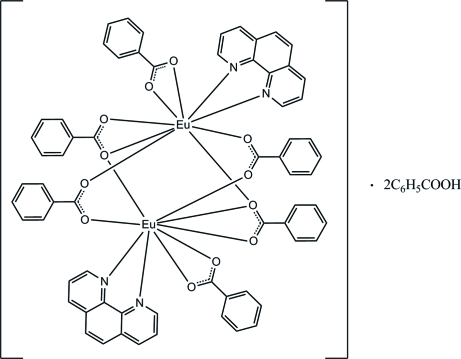


## Experimental

### 

#### Crystal data

[Eu_2_(C_7_H_5_O_2_)_6_(C_12_H_8_N_2_)_2_]·2C_7_H_6_O_2_
*M* *_r_* = 1635.22
Triclinic, 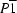

*a* = 9.6688 (2) Å
*b* = 12.8260 (2) Å
*c* = 15.1460 (3) Å
α = 75.244 (1)°
β = 78.605 (1)°
γ = 76.072 (1)°
*V* = 1744.26 (6) Å^3^
*Z* = 1
Mo *K*α radiation
μ = 1.86 mm^−1^
*T* = 296 K
0.58 × 0.32 × 0.28 mm

#### Data collection

Bruker APEXII DUO CCD area-detector diffractometer
Absorption correction: multi-scan (*SADABS*; Bruker, 2009 ▶) *T*_min_ = 0.413, *T*_max_ = 0.628
51513 measured reflections
12557 independent reflections
11674 reflections with *I* > 2σ(*I*)
*R*_int_ = 0.027

#### Refinement

*R*[*F* ^2^ > 2σ(*F* ^2^)] = 0.020
*wR*(*F* ^2^) = 0.050
*S* = 1.04
12557 reflections
464 parameters
H atoms treated by a mixture of independent and constrained refinement
Δρ_max_ = 0.62 e Å^−3^
Δρ_min_ = −0.44 e Å^−3^

### 

Data collection: *APEX2* (Bruker, 2009 ▶); cell refinement: *SAINT* (Bruker, 2009 ▶); data reduction: *SAINT*; program(s) used to solve structure: *SHELXTL* (Sheldrick, 2008 ▶); program(s) used to refine structure: *SHELXTL*; molecular graphics: *SHELXTL*; software used to prepare material for publication: *SHELXTL* and *PLATON* (Spek, 2009 ▶).

## Supplementary Material

Crystal structure: contains datablocks global, I. DOI: 10.1107/S1600536810015229/rz2435sup1.cif

Structure factors: contains datablocks I. DOI: 10.1107/S1600536810015229/rz2435Isup2.hkl

Additional supplementary materials:  crystallographic information; 3D view; checkCIF report

## Figures and Tables

**Table 1:** Selected bond lengths (Å)

Eu1—O5	2.3667 (9)
Eu1—O3^i^	2.3669 (9)
Eu1—O6^i^	2.3818 (10)
Eu1—O4	2.4571 (11)
Eu1—O2	2.4933 (11)
Eu1—O1	2.4974 (11)
Eu1—N1	2.5636 (11)
Eu1—N2	2.6134 (11)
Eu1—O3	2.6394 (11)

Symmetry code: (i) 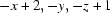.

**Table 2:** Hydrogen-bond geometry (Å, °) *Cg*1 is the centroid of the C35–C40 benzene ring.

*D*—H⋯*A*	*D*—H	H⋯*A*	*D*⋯*A*	*D*—H⋯*A*
O8—H1*O*8⋯O1	0.86 (3)	1.82 (3)	2.660 (2)	166 (3)
C2—H2*A*⋯O6^i^	0.93	2.43	3.017 (2)	121
C9—H9*A*⋯O2^ii^	0.93	2.51	3.258 (2)	138
C11—H11*A*⋯O5	0.93	2.44	3.079 (2)	126
C25—H25*A*⋯*Cg*1^iii^	0.93	2.65	3.551 (3)	164

Symmetry codes: (i) 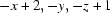; (ii) 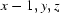; (iii) 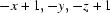.

**Fig. 1.**
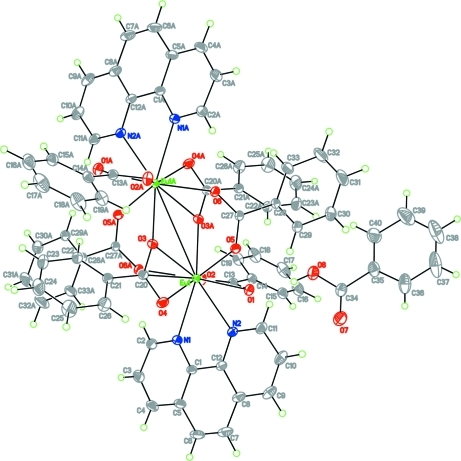
The molecular structure of the title complex, showing 20% probability displacement ellipsoids for non-H atoms and the atom-numbering scheme. The suffix A corresponds to the symmetry code [-x+2, -y, -z+1].

**Fig. 2.**
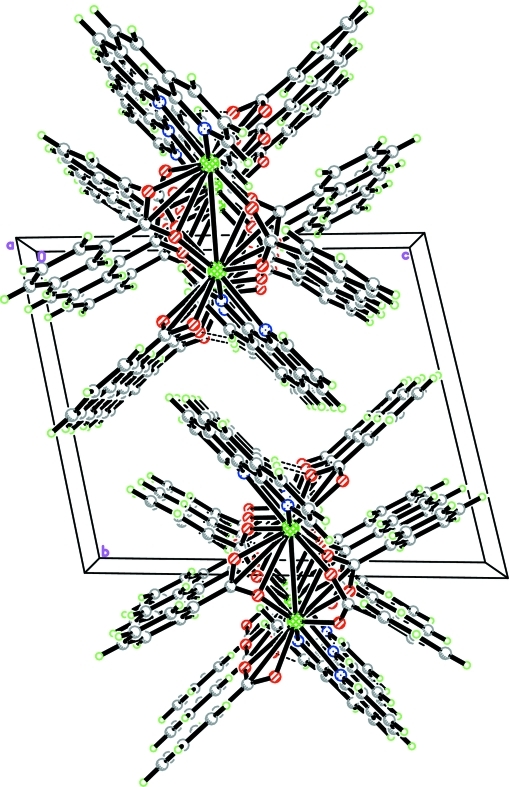
The crystal structure of the title complex, viewed along the a axis, showing two chains along a axis. The benzoic acid solvent molecules have been omitted for clarity. Intermolecular hydrogen bonds are shown as dashed lines.

## supplementary crystallographic information

### Comment

Lanthanide complexes were widely used in biomedical field (Yam & Lo, 1999), sensing areas (Beeby *et al.*, 2003) and optical imaging (Faulkner *et al.*, 2005) because they exhibit intense luminescence, especially europium (III) and terbium (III) ions. In order to create high luminescence complexes, ligands which chelate to the lanthanide atoms should be able to protect lanthanide ions from solvent molecules and absorb energy to transfer it efficiently to the center metal atom (Tang *et al.*, 2008). The title compound, (I) was synthesized and its structure was determined. Similar crystal structures with different lanthanides have been reported in the past, such as lanthanum(III) (Shi *et al.*, 2001), samarium(III) (Niu *et al.*, 1999), gadolinium(III) (Niu *et al.*, 2002) and neodymium(III) (Ooi *et al.*, 2010).

The asymmetric unit of the title complex (I) (Fig. 1) consists of one-half of the complex molecule and one benzoic acid. The complex molecule lies on a crystallographic inversion center. The two europium(III) ions are linked by four benzoate ions, with an Eu—Eu distance of 3.96041 (12) Å. Among the four benzoate ions, two of them also behave as chelating ligands to the europium(III) ions. Each metal ion is coordinated by one phenanthroline heterocycle and a bidentate benzoate ion. The irregular nine-coordinated geometry of the europium(III) ion is completed by seven benzoate O atoms and two phenanthroline N atoms. Bond lengths of Eu—O and Eu—N are listed in Table 1. All other bond lengths and angles are comparable to a closely La-benzoato complex (Shi *et al.*, 2001).

In the crystal structure, intermolecular C9—H9A···O2 hydrogen bonds (Table 2) link the molecules into chains along the *a* axis (Fig. 2). The crystal structure is further stabilized by intermolecular O8—H1O8···O1 and C25A—H25A···*Cg*1 interactions (Table 2). Intramolecular C2—H2A···O6 and C11—H11A···O5 hydrogen bonds (Table 2) stabilize the molecular structure. Weak π–π interactions of *Cg*2···*Cg*3 = 3.6963 (10) Å and *Cg*3···*Cg*3 = 3.6962 (10) Å [*Cg*2 and *Cg*3 are centroids of benzene rings C1/N1/C2–C5 and C1/C5–C8/C12; symmetry code: 1-x, 1-y, 1-z] are observed.

### Experimental

0.5 mmol of EuCl_3_.6H_2_O was dissolved in methanol and then was added into a solution (methanol-H_2_O, 1.5:1) of 1,10-phenanthroline (0.5 mmol) and benzoic acid (1.5 mmol). The mixture was sealed in a tube, and heated directly to 403 K. After keeping at 403 K for 2 days, it was cooled to room temperature. Colourless block crystals (I) were obtained by filtration, and was washed with water and ethanol.

### Refinement

The H1O8 hydrogen atom was located from difference Fourier map and refined freely. All aromatic hydrogen atoms were placed in their calculated positions, with C—H = 0.93 Å, and refined using a riding model with *U*_iso_ = 1.2 *U*_eq_(C).

### Crystal data

**Table:**

[Eu_2_(C_7_H_5_O_2_)_6_(C_12_H_8_N_2_)_2_]·2C_7_H_6_O_2_	*Z* = 1
*M**_r_* = 1635.22	*F*(000) = 820
Triclinic, *P*1	*D*_x_ = 1.557 Mg m^−^^3^
Hall symbol: -P 1	Mo *K*α radiation, λ = 0.71073 Å
*a* = 9.6688 (2) Å	Cell parameters from 9857 reflections
*b* = 12.8260 (2) Å	θ = 2.2–35.2°
*c* = 15.1460 (3) Å	µ = 1.86 mm^−^^1^
α = 75.244 (1)°	*T* = 296 K
β = 78.605 (1)°	Block, colourless
γ = 76.072 (1)°	0.58 × 0.32 × 0.28 mm
*V* = 1744.26 (6) Å^3^	

### Data collection

**Table:**

Bruker APEXII DUO CCD area-detector diffractometer	12557 independent reflections
Radiation source: fine-focus sealed tube	11674 reflections with *I* > 2σ(*I*)
graphite	*R*_int_ = 0.027
φ and ω scans	θ_max_ = 32.5°, θ_min_ = 1.7°
Absorption correction: multi-scan (*SADABS*; Bruker, 2009)	*h* = −14→14
*T*_min_ = 0.413, *T*_max_ = 0.628	*k* = −19→19
51513 measured reflections	*l* = −22→22

### Refinement

**Table:**

Refinement on *F*^2^	Primary atom site location: structure-invariant direct methods
Least-squares matrix: full	Secondary atom site location: difference Fourier map
*R*[*F*^2^ > 2σ(*F*^2^)] = 0.020	Hydrogen site location: inferred from neighbouring sites
*wR*(*F*^2^) = 0.050	H atoms treated by a mixture of independent and constrained refinement
*S* = 1.04	*w* = 1/[σ^2^(*F*_o_^2^) + (0.0249*P*)^2^ + 0.3553*P*] where *P* = (*F*_o_^2^ + 2*F*_c_^2^)/3
12557 reflections	(Δ/σ)_max_ = 0.004
464 parameters	Δρ_max_ = 0.62 e Å^−^^3^
0 restraints	Δρ_min_ = −0.44 e Å^−^^3^

### Special details

**Table:**

Geometry. All esds (except the esd in the dihedral angle between two l.s. planes) are estimated using the full covariance matrix. The cell esds are taken into account individually in the estimation of esds in distances, angles and torsion angles; correlations between esds in cell parameters are only used when they are defined by crystal symmetry. An approximate (isotropic) treatment of cell esds is used for estimating esds involving l.s. planes.
Refinement. Refinement of F^2^ against ALL reflections. The weighted R-factor wR and goodness of fit S are based on F^2^, conventional R-factors R are based on F, with F set to zero for negative F^2^. The threshold expression of F^2^ > 2sigma(F^2^) is used only for calculating R-factors(gt) etc. and is not relevant to the choice of reflections for refinement. R-factors based on F^2^ are statistically about twice as large as those based on F, and R- factors based on ALL data will be even larger.

### Fractional atomic coordinates and isotropic or equivalent isotropic displacement parameters (Å^2^)

**Table:**

	*x*	*y*	*z*	*U*_iso_*/*U*_eq_	
Eu1	0.855968 (6)	0.139628 (5)	0.482146 (4)	0.02529 (2)	
O1	0.81802 (11)	0.27751 (9)	0.33491 (7)	0.0374 (2)	
O2	0.99095 (13)	0.28905 (10)	0.40393 (8)	0.0415 (2)	
O3	0.92345 (10)	−0.05431 (9)	0.59329 (7)	0.0340 (2)	
O4	0.71990 (11)	0.05713 (10)	0.62752 (8)	0.0431 (2)	
O5	0.79737 (11)	0.01384 (8)	0.41205 (7)	0.0357 (2)	
O6	0.98042 (11)	−0.13176 (8)	0.41650 (7)	0.0360 (2)	
N1	0.73487 (13)	0.30002 (10)	0.56106 (9)	0.0348 (2)	
N2	0.58048 (12)	0.21295 (10)	0.47649 (8)	0.0332 (2)	
C1	0.58983 (15)	0.33702 (11)	0.56896 (10)	0.0339 (3)	
C2	0.8100 (2)	0.34414 (14)	0.60131 (13)	0.0450 (4)	
H2A	0.9095	0.3203	0.5951	0.054*	
C3	0.7467 (2)	0.42462 (15)	0.65248 (14)	0.0565 (5)	
H3A	0.8033	0.4523	0.6804	0.068*	
C4	0.6018 (2)	0.46191 (15)	0.66108 (13)	0.0549 (5)	
H4A	0.5581	0.5152	0.6951	0.066*	
C5	0.51860 (19)	0.41920 (13)	0.61810 (11)	0.0446 (4)	
C6	0.3666 (2)	0.45696 (16)	0.62105 (14)	0.0586 (5)	
H6A	0.3195	0.5114	0.6533	0.070*	
C7	0.2904 (2)	0.41550 (17)	0.57823 (15)	0.0580 (5)	
H7A	0.1914	0.4413	0.5818	0.070*	
C8	0.35873 (16)	0.33214 (14)	0.52707 (12)	0.0438 (3)	
C9	0.28376 (17)	0.28860 (16)	0.47982 (14)	0.0532 (4)	
H9A	0.1850	0.3134	0.4805	0.064*	
C10	0.35619 (19)	0.20981 (16)	0.43288 (15)	0.0538 (4)	
H10A	0.3076	0.1802	0.4011	0.065*	
C11	0.50501 (17)	0.17374 (14)	0.43291 (13)	0.0443 (3)	
H11A	0.5533	0.1195	0.4007	0.053*	
C12	0.50850 (15)	0.29219 (11)	0.52310 (10)	0.0341 (3)	
C13	0.92857 (15)	0.31615 (11)	0.33417 (10)	0.0331 (3)	
C14	0.98440 (17)	0.39252 (12)	0.25009 (10)	0.0369 (3)	
C15	0.8962 (2)	0.45586 (14)	0.18589 (11)	0.0464 (4)	
H15A	0.7999	0.4508	0.1943	0.056*	
C16	0.9523 (3)	0.52675 (17)	0.10903 (13)	0.0637 (6)	
H16A	0.8929	0.5704	0.0665	0.076*	
C17	1.0955 (3)	0.5327 (2)	0.09555 (15)	0.0733 (7)	
H17A	1.1322	0.5805	0.0439	0.088*	
C18	1.1843 (3)	0.4690 (2)	0.15733 (14)	0.0650 (6)	
H18A	1.2813	0.4725	0.1471	0.078*	
C19	1.1292 (2)	0.39925 (16)	0.23535 (12)	0.0488 (4)	
H19A	1.1891	0.3568	0.2780	0.059*	
C20	0.81636 (14)	−0.02422 (11)	0.65167 (9)	0.0312 (2)	
C21	0.80887 (15)	−0.08375 (12)	0.75001 (10)	0.0344 (3)	
C22	0.93267 (18)	−0.12514 (15)	0.79046 (11)	0.0444 (3)	
H22A	1.0219	−0.1178	0.7556	0.053*	
C23	0.9241 (3)	−0.1774 (2)	0.88256 (13)	0.0621 (5)	
H23A	1.0076	−0.2050	0.9097	0.074*	
C24	0.7932 (3)	−0.1885 (2)	0.93387 (14)	0.0716 (6)	
H24A	0.7881	−0.2243	0.9957	0.086*	
C25	0.6697 (3)	−0.1473 (2)	0.89483 (15)	0.0732 (7)	
H25A	0.5810	−0.1548	0.9304	0.088*	
C26	0.6760 (2)	−0.09452 (18)	0.80281 (13)	0.0542 (4)	
H26A	0.5920	−0.0664	0.7764	0.065*	
C27	0.86800 (14)	−0.07267 (11)	0.38672 (9)	0.0298 (2)	
C28	0.81424 (15)	−0.11023 (12)	0.31629 (10)	0.0340 (3)	
C29	0.66775 (17)	−0.09528 (14)	0.31412 (11)	0.0404 (3)	
H29A	0.6017	−0.0556	0.3532	0.048*	
C30	0.6193 (2)	−0.13920 (18)	0.25387 (14)	0.0554 (5)	
H30A	0.5211	−0.1313	0.2541	0.066*	
C31	0.7167 (3)	−0.1941 (3)	0.19409 (17)	0.0785 (8)	
H31A	0.6842	−0.2232	0.1535	0.094*	
C32	0.8625 (3)	−0.2068 (3)	0.19349 (18)	0.0872 (9)	
H32A	0.9279	−0.2428	0.1516	0.105*	
C33	0.9119 (2)	−0.16586 (19)	0.25529 (14)	0.0583 (5)	
H33A	1.0103	−0.1757	0.2558	0.070*	
O7	0.5814 (2)	0.39064 (16)	0.15565 (15)	0.0873 (6)	
O8	0.72156 (19)	0.22869 (14)	0.20067 (12)	0.0695 (4)	
C34	0.6441 (2)	0.30199 (19)	0.14202 (15)	0.0574 (5)	
C35	0.6454 (2)	0.2640 (2)	0.05692 (15)	0.0620 (5)	
C36	0.5708 (3)	0.3347 (3)	−0.01171 (18)	0.0841 (9)	
H36A	0.5192	0.4036	−0.0033	0.101*	
C37	0.5733 (5)	0.3028 (4)	−0.0927 (2)	0.1221 (17)	
H37A	0.5222	0.3501	−0.1382	0.147*	
C38	0.6484 (5)	0.2046 (5)	−0.1061 (3)	0.135 (2)	
H38A	0.6505	0.1849	−0.1615	0.162*	
C39	0.7221 (4)	0.1329 (4)	−0.0395 (3)	0.1310 (17)	
H39A	0.7730	0.0643	−0.0492	0.157*	
C40	0.7205 (3)	0.1630 (3)	0.0436 (2)	0.0896 (9)	
H40A	0.7703	0.1146	0.0893	0.108*	
H1O8	0.743 (3)	0.255 (2)	0.242 (2)	0.089 (9)*	

### Atomic displacement parameters (Å^2^)

**Table:**

	*U*^11^	*U*^22^	*U*^33^	*U*^12^	*U*^13^	*U*^23^
Eu1	0.02319 (3)	0.02625 (3)	0.02639 (3)	−0.00168 (2)	−0.00573 (2)	−0.00710 (2)
O1	0.0346 (5)	0.0412 (5)	0.0364 (5)	−0.0105 (4)	−0.0090 (4)	−0.0030 (4)
O2	0.0442 (6)	0.0451 (6)	0.0377 (5)	−0.0170 (5)	−0.0131 (4)	−0.0005 (5)
O3	0.0301 (4)	0.0394 (5)	0.0311 (5)	−0.0039 (4)	0.0004 (4)	−0.0116 (4)
O4	0.0322 (5)	0.0463 (6)	0.0375 (5)	0.0027 (4)	0.0008 (4)	0.0001 (5)
O5	0.0316 (5)	0.0362 (5)	0.0440 (5)	−0.0021 (4)	−0.0116 (4)	−0.0169 (4)
O6	0.0349 (5)	0.0360 (5)	0.0400 (5)	−0.0001 (4)	−0.0161 (4)	−0.0119 (4)
N1	0.0361 (6)	0.0325 (5)	0.0369 (6)	−0.0022 (4)	−0.0066 (5)	−0.0132 (5)
N2	0.0277 (5)	0.0329 (5)	0.0383 (6)	−0.0033 (4)	−0.0075 (4)	−0.0072 (5)
C1	0.0352 (6)	0.0286 (6)	0.0320 (6)	0.0005 (5)	−0.0016 (5)	−0.0052 (5)
C2	0.0497 (9)	0.0391 (8)	0.0524 (9)	−0.0029 (7)	−0.0176 (7)	−0.0190 (7)
C3	0.0783 (13)	0.0440 (9)	0.0559 (10)	−0.0044 (9)	−0.0228 (10)	−0.0236 (8)
C4	0.0786 (13)	0.0392 (8)	0.0440 (9)	0.0030 (8)	−0.0062 (8)	−0.0198 (7)
C5	0.0518 (9)	0.0349 (7)	0.0379 (7)	0.0034 (6)	0.0028 (6)	−0.0098 (6)
C6	0.0545 (10)	0.0489 (10)	0.0546 (10)	0.0116 (8)	0.0107 (8)	−0.0147 (8)
C7	0.0361 (8)	0.0528 (10)	0.0656 (12)	0.0086 (7)	0.0078 (8)	−0.0065 (9)
C8	0.0286 (6)	0.0411 (8)	0.0496 (9)	−0.0011 (6)	0.0005 (6)	0.0013 (6)
C9	0.0273 (7)	0.0531 (10)	0.0701 (12)	−0.0062 (7)	−0.0110 (7)	0.0043 (9)
C10	0.0367 (8)	0.0533 (10)	0.0745 (13)	−0.0119 (7)	−0.0241 (8)	−0.0048 (9)
C11	0.0358 (7)	0.0435 (8)	0.0576 (10)	−0.0052 (6)	−0.0176 (7)	−0.0125 (7)
C12	0.0280 (6)	0.0313 (6)	0.0363 (7)	−0.0017 (5)	−0.0016 (5)	−0.0020 (5)
C13	0.0346 (6)	0.0299 (6)	0.0337 (6)	−0.0051 (5)	−0.0040 (5)	−0.0069 (5)
C14	0.0469 (8)	0.0322 (6)	0.0326 (6)	−0.0115 (6)	−0.0018 (6)	−0.0084 (5)
C15	0.0595 (10)	0.0405 (8)	0.0358 (7)	−0.0071 (7)	−0.0066 (7)	−0.0052 (6)
C16	0.0993 (18)	0.0486 (10)	0.0379 (9)	−0.0153 (11)	−0.0103 (10)	0.0004 (8)
C17	0.111 (2)	0.0694 (14)	0.0427 (10)	−0.0494 (14)	0.0106 (11)	−0.0050 (9)
C18	0.0762 (14)	0.0797 (15)	0.0493 (10)	−0.0467 (12)	0.0072 (10)	−0.0152 (10)
C19	0.0532 (9)	0.0556 (10)	0.0434 (8)	−0.0253 (8)	−0.0025 (7)	−0.0112 (7)
C20	0.0272 (5)	0.0340 (6)	0.0310 (6)	−0.0067 (5)	−0.0010 (5)	−0.0067 (5)
C21	0.0348 (6)	0.0347 (6)	0.0307 (6)	−0.0067 (5)	−0.0007 (5)	−0.0050 (5)
C22	0.0399 (8)	0.0537 (9)	0.0361 (7)	−0.0031 (7)	−0.0069 (6)	−0.0084 (7)
C23	0.0649 (12)	0.0734 (14)	0.0403 (9)	−0.0016 (10)	−0.0174 (8)	−0.0029 (9)
C24	0.0862 (16)	0.0812 (16)	0.0349 (9)	−0.0182 (13)	−0.0055 (10)	0.0082 (9)
C25	0.0634 (13)	0.0953 (18)	0.0467 (11)	−0.0302 (13)	0.0106 (9)	0.0090 (11)
C26	0.0387 (8)	0.0701 (12)	0.0456 (9)	−0.0165 (8)	0.0015 (7)	0.0014 (8)
C27	0.0294 (6)	0.0320 (6)	0.0302 (6)	−0.0059 (5)	−0.0079 (5)	−0.0082 (5)
C28	0.0350 (6)	0.0371 (7)	0.0333 (6)	−0.0040 (5)	−0.0107 (5)	−0.0122 (5)
C29	0.0375 (7)	0.0447 (8)	0.0442 (8)	−0.0051 (6)	−0.0137 (6)	−0.0160 (6)
C30	0.0492 (9)	0.0703 (12)	0.0596 (11)	−0.0131 (9)	−0.0228 (8)	−0.0250 (10)
C31	0.0720 (14)	0.117 (2)	0.0725 (15)	−0.0194 (14)	−0.0213 (12)	−0.0584 (15)
C32	0.0660 (14)	0.139 (3)	0.0799 (16)	−0.0072 (15)	−0.0076 (12)	−0.0808 (18)
C33	0.0419 (9)	0.0859 (14)	0.0565 (11)	−0.0017 (9)	−0.0077 (8)	−0.0421 (11)
O7	0.0881 (13)	0.0763 (12)	0.1062 (15)	0.0128 (10)	−0.0490 (11)	−0.0364 (11)
O8	0.0750 (10)	0.0691 (10)	0.0727 (10)	0.0023 (8)	−0.0379 (8)	−0.0250 (8)
C34	0.0493 (10)	0.0676 (13)	0.0621 (12)	−0.0123 (9)	−0.0194 (9)	−0.0168 (10)
C35	0.0574 (11)	0.0837 (15)	0.0582 (11)	−0.0341 (11)	−0.0111 (9)	−0.0183 (11)
C36	0.103 (2)	0.101 (2)	0.0633 (14)	−0.0613 (18)	−0.0300 (14)	0.0085 (13)
C37	0.155 (4)	0.191 (4)	0.0526 (15)	−0.121 (4)	−0.0252 (19)	0.006 (2)
C38	0.127 (3)	0.246 (6)	0.083 (2)	−0.108 (4)	0.012 (2)	−0.081 (3)
C39	0.090 (2)	0.201 (5)	0.149 (4)	−0.038 (3)	−0.005 (2)	−0.123 (4)
C40	0.0670 (15)	0.119 (2)	0.107 (2)	−0.0156 (15)	−0.0216 (15)	−0.063 (2)

### Geometric parameters (Å, °)

**Table:**

Eu1—O5	2.3667 (9)	C15—H15A	0.9300
Eu1—O3^i^	2.3669 (9)	C16—C17	1.377 (4)
Eu1—O6^i^	2.3818 (10)	C16—H16A	0.9300
Eu1—O4	2.4571 (11)	C17—C18	1.368 (4)
Eu1—O2	2.4933 (11)	C17—H17A	0.9300
Eu1—O1	2.4974 (11)	C18—C19	1.387 (3)
Eu1—N1	2.5636 (11)	C18—H18A	0.9300
Eu1—N2	2.6134 (11)	C19—H19A	0.9300
Eu1—O3	2.6394 (11)	C20—C21	1.487 (2)
Eu1—C13	2.8637 (14)	C21—C22	1.381 (2)
Eu1—C20	2.8992 (14)	C21—C26	1.390 (2)
Eu1—Eu1^i^	3.96041 (12)	C22—C23	1.381 (3)
O1—C13	1.2789 (18)	C22—H22A	0.9300
O2—C13	1.2509 (18)	C23—C24	1.367 (3)
O3—C20	1.2739 (15)	C23—H23A	0.9300
O3—Eu1^i^	2.3668 (9)	C24—C25	1.368 (4)
O4—C20	1.2506 (17)	C24—H24A	0.9300
O5—C27	1.2623 (16)	C25—C26	1.382 (3)
O6—C27	1.2579 (16)	C25—H25A	0.9300
O6—Eu1^i^	2.3819 (10)	C26—H26A	0.9300
N1—C2	1.330 (2)	C27—C28	1.4971 (18)
N1—C1	1.3596 (18)	C28—C33	1.386 (2)
N2—C11	1.3256 (19)	C28—C29	1.389 (2)
N2—C12	1.3589 (18)	C29—C30	1.388 (2)
C1—C5	1.4096 (19)	C29—H29A	0.9300
C1—C12	1.438 (2)	C30—C31	1.368 (3)
C2—C3	1.399 (2)	C30—H30A	0.9300
C2—H2A	0.9300	C31—C32	1.379 (3)
C3—C4	1.359 (3)	C31—H31A	0.9300
C3—H3A	0.9300	C32—C33	1.389 (3)
C4—C5	1.404 (3)	C32—H32A	0.9300
C4—H4A	0.9300	C33—H33A	0.9300
C5—C6	1.428 (3)	O7—C34	1.200 (3)
C6—C7	1.341 (3)	O8—C34	1.316 (3)
C6—H6A	0.9300	O8—H1O8	0.86 (3)
C7—C8	1.435 (3)	C34—C35	1.486 (3)
C7—H7A	0.9300	C35—C40	1.366 (4)
C8—C9	1.397 (3)	C35—C36	1.387 (4)
C8—C12	1.410 (2)	C36—C37	1.383 (4)
C9—C10	1.359 (3)	C36—H36A	0.9300
C9—H9A	0.9300	C37—C38	1.336 (7)
C10—C11	1.401 (2)	C37—H37A	0.9300
C10—H10A	0.9300	C38—C39	1.367 (7)
C11—H11A	0.9300	C38—H38A	0.9300
C13—C14	1.490 (2)	C39—C40	1.405 (4)
C14—C15	1.387 (2)	C39—H39A	0.9300
C14—C19	1.394 (2)	C40—H40A	0.9300
C15—C16	1.387 (3)		
			
O5—Eu1—O3^i^	74.07 (3)	C10—C9—C8	119.53 (15)
O5—Eu1—O6^i^	136.23 (4)	C10—C9—H9A	120.2
O3^i^—Eu1—O6^i^	78.20 (3)	C8—C9—H9A	120.2
O5—Eu1—O4	88.22 (4)	C9—C10—C11	119.19 (17)
O3^i^—Eu1—O4	126.28 (4)	C9—C10—H10A	120.4
O6^i^—Eu1—O4	81.61 (4)	C11—C10—H10A	120.4
O5—Eu1—O2	126.55 (4)	N2—C11—C10	123.32 (17)
O3^i^—Eu1—O2	73.68 (4)	N2—C11—H11A	118.3
O6^i^—Eu1—O2	74.81 (4)	C10—C11—H11A	118.3
O4—Eu1—O2	145.08 (4)	N2—C12—C8	122.47 (14)
O5—Eu1—O1	86.23 (4)	N2—C12—C1	118.12 (12)
O3^i^—Eu1—O1	89.18 (4)	C8—C12—C1	119.41 (14)
O6^i^—Eu1—O1	126.75 (4)	O2—C13—O1	119.97 (13)
O4—Eu1—O1	140.76 (4)	O2—C13—C14	119.79 (13)
O2—Eu1—O1	52.07 (3)	O1—C13—C14	120.23 (13)
O5—Eu1—N1	140.57 (4)	O2—C13—Eu1	60.31 (8)
O3^i^—Eu1—N1	144.07 (4)	O1—C13—Eu1	60.57 (7)
O6^i^—Eu1—N1	76.95 (4)	C14—C13—Eu1	169.95 (10)
O4—Eu1—N1	74.78 (4)	C15—C14—C19	119.41 (15)
O2—Eu1—N1	75.04 (4)	C15—C14—C13	121.60 (15)
O1—Eu1—N1	85.33 (4)	C19—C14—C13	118.98 (15)
O5—Eu1—N2	77.50 (4)	C16—C15—C14	119.73 (19)
O3^i^—Eu1—N2	145.74 (4)	C16—C15—H15A	120.1
O6^i^—Eu1—N2	136.06 (4)	C14—C15—H15A	120.1
O4—Eu1—N2	70.77 (4)	C17—C16—C15	120.2 (2)
O2—Eu1—N2	110.01 (4)	C17—C16—H16A	119.9
O1—Eu1—N2	70.10 (4)	C15—C16—H16A	119.9
N1—Eu1—N2	63.41 (4)	C18—C17—C16	120.69 (19)
O5—Eu1—O3	75.18 (3)	C18—C17—H17A	119.7
O3^i^—Eu1—O3	75.56 (4)	C16—C17—H17A	119.7
O6^i^—Eu1—O3	65.45 (3)	C17—C18—C19	119.8 (2)
O4—Eu1—O3	50.80 (3)	C17—C18—H18A	120.1
O2—Eu1—O3	133.58 (3)	C19—C18—H18A	120.1
O1—Eu1—O3	158.51 (3)	C18—C19—C14	120.20 (19)
N1—Eu1—O3	115.90 (3)	C18—C19—H19A	119.9
N2—Eu1—O3	115.03 (3)	C14—C19—H19A	119.9
O5—Eu1—C13	105.75 (4)	O4—C20—O3	120.53 (13)
O3^i^—Eu1—C13	77.90 (4)	O4—C20—C21	119.75 (12)
O6^i^—Eu1—C13	100.63 (4)	O3—C20—C21	119.68 (12)
O4—Eu1—C13	155.22 (4)	O4—C20—Eu1	57.16 (7)
O2—Eu1—C13	25.84 (4)	O3—C20—Eu1	65.51 (7)
O1—Eu1—C13	26.49 (4)	C21—C20—Eu1	162.76 (10)
N1—Eu1—C13	81.65 (4)	C22—C21—C26	119.52 (15)
N2—Eu1—C13	91.99 (4)	C22—C21—C20	120.52 (13)
O3—Eu1—C13	152.04 (3)	C26—C21—C20	119.92 (14)
O5—Eu1—C20	84.79 (4)	C23—C22—C21	120.07 (17)
O3^i^—Eu1—C20	101.47 (4)	C23—C22—H22A	120.0
O6^i^—Eu1—C20	68.28 (4)	C21—C22—H22A	120.0
O4—Eu1—C20	25.32 (4)	C24—C23—C22	120.1 (2)
O2—Eu1—C20	142.92 (4)	C24—C23—H23A	120.0
O1—Eu1—C20	163.61 (4)	C22—C23—H23A	120.0
N1—Eu1—C20	92.95 (4)	C23—C24—C25	120.45 (19)
N2—Eu1—C20	94.54 (4)	C23—C24—H24A	119.8
O3—Eu1—C20	26.05 (3)	C25—C24—H24A	119.8
C13—Eu1—C20	168.62 (4)	C24—C25—C26	120.28 (19)
O5—Eu1—Eu1^i^	70.46 (2)	C24—C25—H25A	119.9
O3^i^—Eu1—Eu1^i^	40.20 (3)	C26—C25—H25A	119.9
O6^i^—Eu1—Eu1^i^	66.47 (2)	C25—C26—C21	119.59 (19)
O4—Eu1—Eu1^i^	86.12 (3)	C25—C26—H26A	120.2
O2—Eu1—Eu1^i^	106.95 (3)	C21—C26—H26A	120.2
O1—Eu1—Eu1^i^	127.70 (3)	O6—C27—O5	125.43 (12)
N1—Eu1—Eu1^i^	140.81 (3)	O6—C27—C28	116.39 (12)
N2—Eu1—Eu1^i^	140.89 (3)	O5—C27—C28	118.18 (12)
O3—Eu1—Eu1^i^	35.36 (2)	C33—C28—C29	119.37 (14)
C13—Eu1—Eu1^i^	117.61 (3)	C33—C28—C27	119.47 (13)
C20—Eu1—Eu1^i^	61.32 (3)	C29—C28—C27	121.07 (13)
C13—O1—Eu1	92.94 (9)	C30—C29—C28	120.35 (15)
C13—O2—Eu1	93.86 (9)	C30—C29—H29A	119.8
C20—O3—Eu1^i^	165.77 (10)	C28—C29—H29A	119.8
C20—O3—Eu1	88.44 (8)	C31—C30—C29	119.75 (18)
Eu1^i^—O3—Eu1	104.44 (4)	C31—C30—H30A	120.1
C20—O4—Eu1	97.52 (8)	C29—C30—H30A	120.1
C27—O5—Eu1	133.44 (9)	C30—C31—C32	120.57 (18)
C27—O6—Eu1^i^	141.23 (9)	C30—C31—H31A	119.7
C2—N1—C1	117.91 (13)	C32—C31—H31A	119.7
C2—N1—Eu1	121.18 (10)	C31—C32—C33	120.08 (19)
C1—N1—Eu1	120.74 (9)	C31—C32—H32A	120.0
C11—N2—C12	117.65 (13)	C33—C32—H32A	120.0
C11—N2—Eu1	123.20 (10)	C28—C33—C32	119.82 (18)
C12—N2—Eu1	119.10 (9)	C28—C33—H33A	120.1
N1—C1—C5	122.16 (14)	C32—C33—H33A	120.1
N1—C1—C12	118.18 (12)	C34—O8—H1O8	114 (2)
C5—C1—C12	119.63 (14)	O7—C34—O8	123.2 (2)
N1—C2—C3	123.21 (17)	O7—C34—C35	124.0 (2)
N1—C2—H2A	118.4	O8—C34—C35	112.8 (2)
C3—C2—H2A	118.4	C40—C35—C36	119.4 (2)
C4—C3—C2	119.36 (17)	C40—C35—C34	122.0 (2)
C4—C3—H3A	120.3	C36—C35—C34	118.6 (2)
C2—C3—H3A	120.3	C37—C36—C35	119.9 (4)
C3—C4—C5	119.34 (15)	C37—C36—H36A	120.0
C3—C4—H4A	120.3	C35—C36—H36A	120.0
C5—C4—H4A	120.3	C38—C37—C36	120.6 (4)
C4—C5—C1	118.01 (16)	C38—C37—H37A	119.7
C4—C5—C6	122.71 (16)	C36—C37—H37A	119.7
C1—C5—C6	119.28 (17)	C37—C38—C39	120.7 (3)
C7—C6—C5	121.27 (16)	C37—C38—H38A	119.6
C7—C6—H6A	119.4	C39—C38—H38A	119.6
C5—C6—H6A	119.4	C38—C39—C40	119.7 (4)
C6—C7—C8	121.27 (16)	C38—C39—H39A	120.1
C6—C7—H7A	119.4	C40—C39—H39A	120.1
C8—C7—H7A	119.4	C35—C40—C39	119.6 (4)
C9—C8—C12	117.84 (16)	C35—C40—H40A	120.2
C9—C8—C7	123.03 (16)	C39—C40—H40A	120.2
C12—C8—C7	119.12 (17)		
			
O5—Eu1—O1—C13	−137.91 (9)	C9—C8—C12—C1	178.47 (15)
O3^i^—Eu1—O1—C13	−63.82 (8)	C7—C8—C12—C1	−1.0 (2)
O6^i^—Eu1—O1—C13	10.75 (10)	N1—C1—C12—N2	1.8 (2)
O4—Eu1—O1—C13	139.55 (9)	C5—C1—C12—N2	−179.89 (13)
O2—Eu1—O1—C13	5.99 (8)	N1—C1—C12—C8	−177.52 (14)
N1—Eu1—O1—C13	80.64 (9)	C5—C1—C12—C8	0.8 (2)
N2—Eu1—O1—C13	144.02 (9)	Eu1—O2—C13—O1	10.94 (14)
O3—Eu1—O1—C13	−108.00 (11)	Eu1—O2—C13—C14	−168.42 (11)
C20—Eu1—O1—C13	165.19 (12)	Eu1—O1—C13—O2	−10.92 (14)
Eu1^i^—Eu1—O1—C13	−76.24 (9)	Eu1—O1—C13—C14	168.44 (11)
O5—Eu1—O2—C13	40.92 (10)	O5—Eu1—C13—O2	−146.86 (9)
O3^i^—Eu1—O2—C13	95.95 (9)	O3^i^—Eu1—C13—O2	−77.47 (9)
O6^i^—Eu1—O2—C13	177.82 (10)	O6^i^—Eu1—C13—O2	−2.14 (9)
O4—Eu1—O2—C13	−132.93 (9)	O4—Eu1—C13—O2	90.88 (13)
O1—Eu1—O2—C13	−6.13 (8)	O1—Eu1—C13—O2	169.12 (14)
N1—Eu1—O2—C13	−101.94 (9)	N1—Eu1—C13—O2	72.81 (9)
N2—Eu1—O2—C13	−48.13 (10)	N2—Eu1—C13—O2	135.56 (9)
O3—Eu1—O2—C13	146.34 (8)	O3—Eu1—C13—O2	−58.89 (12)
C20—Eu1—O2—C13	−176.56 (8)	C20—Eu1—C13—O2	10.6 (2)
Eu1^i^—Eu1—O2—C13	118.83 (8)	Eu1^i^—Eu1—C13—O2	−71.02 (9)
O5—Eu1—O3—C20	−109.12 (8)	O5—Eu1—C13—O1	44.02 (9)
O3^i^—Eu1—O3—C20	173.89 (10)	O3^i^—Eu1—C13—O1	113.41 (9)
O6^i^—Eu1—O3—C20	90.46 (8)	O6^i^—Eu1—C13—O1	−171.25 (8)
O4—Eu1—O3—C20	−9.08 (8)	O4—Eu1—C13—O1	−78.24 (13)
O2—Eu1—O3—C20	124.11 (8)	O2—Eu1—C13—O1	−169.12 (14)
O1—Eu1—O3—C20	−140.10 (10)	N1—Eu1—C13—O1	−96.31 (9)
N1—Eu1—O3—C20	30.32 (9)	N2—Eu1—C13—O1	−33.55 (9)
N2—Eu1—O3—C20	−40.86 (9)	O3—Eu1—C13—O1	131.99 (9)
C13—Eu1—O3—C20	155.12 (9)	C20—Eu1—C13—O1	−158.57 (16)
Eu1^i^—Eu1—O3—C20	173.88 (10)	Eu1^i^—Eu1—C13—O1	119.86 (8)
O5—Eu1—O3—Eu1^i^	77.00 (4)	O5—Eu1—C13—C14	−53.4 (6)
O3^i^—Eu1—O3—Eu1^i^	0.0	O3^i^—Eu1—C13—C14	16.0 (6)
O6^i^—Eu1—O3—Eu1^i^	−83.42 (4)	O6^i^—Eu1—C13—C14	91.3 (6)
O4—Eu1—O3—Eu1^i^	177.04 (6)	O4—Eu1—C13—C14	−175.7 (6)
O2—Eu1—O3—Eu1^i^	−49.77 (6)	O2—Eu1—C13—C14	93.4 (6)
O1—Eu1—O3—Eu1^i^	46.02 (10)	O1—Eu1—C13—C14	−97.4 (6)
N1—Eu1—O3—Eu1^i^	−143.56 (4)	N1—Eu1—C13—C14	166.3 (6)
N2—Eu1—O3—Eu1^i^	145.25 (4)	N2—Eu1—C13—C14	−131.0 (6)
C13—Eu1—O3—Eu1^i^	−18.77 (9)	O3—Eu1—C13—C14	34.6 (6)
C20—Eu1—O3—Eu1^i^	−173.88 (10)	C20—Eu1—C13—C14	104.0 (6)
O5—Eu1—O4—C20	81.57 (9)	Eu1^i^—Eu1—C13—C14	22.4 (6)
O3^i^—Eu1—O4—C20	12.88 (11)	O2—C13—C14—C15	−156.05 (15)
O6^i^—Eu1—O4—C20	−55.73 (9)	O1—C13—C14—C15	24.6 (2)
O2—Eu1—O4—C20	−103.37 (10)	Eu1—C13—C14—C15	116.3 (6)
O1—Eu1—O4—C20	163.41 (8)	O2—C13—C14—C19	24.7 (2)
N1—Eu1—O4—C20	−134.40 (10)	O1—C13—C14—C19	−154.66 (15)
N2—Eu1—O4—C20	158.96 (10)	Eu1—C13—C14—C19	−63.0 (6)
O3—Eu1—O4—C20	9.32 (8)	C19—C14—C15—C16	−1.4 (2)
C13—Eu1—O4—C20	−152.94 (10)	C13—C14—C15—C16	179.34 (15)
Eu1^i^—Eu1—O4—C20	11.04 (9)	C14—C15—C16—C17	1.3 (3)
O3^i^—Eu1—O5—C27	23.71 (13)	C15—C16—C17—C18	0.1 (3)
O6^i^—Eu1—O5—C27	−29.02 (15)	C16—C17—C18—C19	−1.3 (4)
O4—Eu1—O5—C27	−104.93 (13)	C17—C18—C19—C14	1.1 (3)
O2—Eu1—O5—C27	78.58 (14)	C15—C14—C19—C18	0.2 (3)
O1—Eu1—O5—C27	113.93 (13)	C13—C14—C19—C18	179.51 (16)
N1—Eu1—O5—C27	−168.10 (12)	Eu1—O4—C20—O3	−17.58 (15)
N2—Eu1—O5—C27	−175.63 (14)	Eu1—O4—C20—C21	160.12 (11)
O3—Eu1—O5—C27	−55.17 (13)	Eu1^i^—O3—C20—O4	171.4 (3)
C13—Eu1—O5—C27	95.84 (13)	Eu1—O3—C20—O4	16.19 (14)
C20—Eu1—O5—C27	−79.80 (13)	Eu1^i^—O3—C20—C21	−6.3 (5)
Eu1^i^—Eu1—O5—C27	−18.42 (12)	Eu1—O3—C20—C21	−161.51 (12)
O5—Eu1—N1—C2	172.83 (12)	Eu1^i^—O3—C20—Eu1	155.2 (4)
O3^i^—Eu1—N1—C2	−26.78 (16)	O5—Eu1—C20—O4	−96.87 (10)
O6^i^—Eu1—N1—C2	20.54 (13)	O3^i^—Eu1—C20—O4	−169.43 (9)
O4—Eu1—N1—C2	105.26 (13)	O6^i^—Eu1—C20—O4	118.36 (10)
O2—Eu1—N1—C2	−56.95 (13)	O2—Eu1—C20—O4	112.53 (10)
O1—Eu1—N1—C2	−108.89 (13)	O1—Eu1—C20—O4	−39.80 (18)
N2—Eu1—N1—C2	−178.95 (14)	N1—Eu1—C20—O4	43.66 (10)
O3—Eu1—N1—C2	74.62 (13)	N2—Eu1—C20—O4	−19.88 (10)
C13—Eu1—N1—C2	−82.48 (13)	O3—Eu1—C20—O4	−163.39 (14)
C20—Eu1—N1—C2	87.45 (13)	C13—Eu1—C20—O4	104.9 (2)
Eu1^i^—Eu1—N1—C2	41.66 (15)	Eu1^i^—Eu1—C20—O4	−167.42 (10)
O5—Eu1—N1—C1	−2.21 (14)	O5—Eu1—C20—O3	66.52 (8)
O3^i^—Eu1—N1—C1	158.18 (9)	O3^i^—Eu1—C20—O3	−6.04 (10)
O6^i^—Eu1—N1—C1	−154.50 (11)	O6^i^—Eu1—C20—O3	−78.25 (8)
O4—Eu1—N1—C1	−69.78 (11)	O4—Eu1—C20—O3	163.39 (14)
O2—Eu1—N1—C1	128.00 (11)	O2—Eu1—C20—O3	−84.08 (10)
O1—Eu1—N1—C1	76.07 (11)	O1—Eu1—C20—O3	123.60 (13)
N2—Eu1—N1—C1	6.01 (10)	N1—Eu1—C20—O3	−152.95 (8)
O3—Eu1—N1—C1	−100.42 (11)	N2—Eu1—C20—O3	143.51 (8)
C13—Eu1—N1—C1	102.48 (11)	C13—Eu1—C20—O3	−91.7 (2)
C20—Eu1—N1—C1	−87.60 (11)	Eu1^i^—Eu1—C20—O3	−4.03 (7)
Eu1^i^—Eu1—N1—C1	−133.38 (9)	O5—Eu1—C20—C21	178.1 (3)
O5—Eu1—N2—C11	−7.65 (13)	O3^i^—Eu1—C20—C21	105.6 (3)
O3^i^—Eu1—N2—C11	26.80 (16)	O6^i^—Eu1—C20—C21	33.3 (3)
O6^i^—Eu1—N2—C11	−154.38 (12)	O4—Eu1—C20—C21	−85.0 (3)
O4—Eu1—N2—C11	−100.15 (13)	O2—Eu1—C20—C21	27.5 (3)
O2—Eu1—N2—C11	116.99 (13)	O1—Eu1—C20—C21	−124.8 (3)
O1—Eu1—N2—C11	82.84 (13)	N1—Eu1—C20—C21	−41.4 (3)
N1—Eu1—N2—C11	177.69 (14)	N2—Eu1—C20—C21	−104.9 (3)
O3—Eu1—N2—C11	−74.53 (13)	O3—Eu1—C20—C21	111.6 (3)
C13—Eu1—N2—C11	98.04 (13)	C13—Eu1—C20—C21	19.9 (4)
C20—Eu1—N2—C11	−91.29 (13)	Eu1^i^—Eu1—C20—C21	107.6 (3)
Eu1^i^—Eu1—N2—C11	−43.01 (14)	O4—C20—C21—C22	−142.59 (16)
O5—Eu1—N2—C12	169.73 (11)	O3—C20—C21—C22	35.1 (2)
O3^i^—Eu1—N2—C12	−155.82 (9)	Eu1—C20—C21—C22	−68.0 (4)
O6^i^—Eu1—N2—C12	23.01 (13)	O4—C20—C21—C26	35.0 (2)
O4—Eu1—N2—C12	77.23 (10)	O3—C20—C21—C26	−147.24 (16)
O2—Eu1—N2—C12	−65.62 (11)	Eu1—C20—C21—C26	109.6 (3)
O1—Eu1—N2—C12	−99.77 (11)	C26—C21—C22—C23	0.4 (3)
N1—Eu1—N2—C12	−4.93 (10)	C20—C21—C22—C23	178.04 (17)
O3—Eu1—N2—C12	102.85 (10)	C21—C22—C23—C24	0.1 (3)
C13—Eu1—N2—C12	−84.57 (10)	C22—C23—C24—C25	−0.6 (4)
C20—Eu1—N2—C12	86.09 (10)	C23—C24—C25—C26	0.4 (4)
Eu1^i^—Eu1—N2—C12	134.37 (9)	C24—C25—C26—C21	0.1 (4)
C2—N1—C1—C5	−0.2 (2)	C22—C21—C26—C25	−0.5 (3)
Eu1—N1—C1—C5	175.00 (11)	C20—C21—C26—C25	−178.2 (2)
C2—N1—C1—C12	178.03 (14)	Eu1^i^—O6—C27—O5	−3.4 (3)
Eu1—N1—C1—C12	−6.77 (17)	Eu1^i^—O6—C27—C28	177.45 (10)
C1—N1—C2—C3	1.3 (3)	Eu1—O5—C27—O6	21.8 (2)
Eu1—N1—C2—C3	−173.87 (14)	Eu1—O5—C27—C28	−159.07 (10)
N1—C2—C3—C4	−1.1 (3)	O6—C27—C28—C33	−31.8 (2)
C2—C3—C4—C5	−0.2 (3)	O5—C27—C28—C33	148.90 (17)
C3—C4—C5—C1	1.3 (3)	O6—C27—C28—C29	144.80 (15)
C3—C4—C5—C6	−177.65 (19)	O5—C27—C28—C29	−34.4 (2)
N1—C1—C5—C4	−1.1 (2)	C33—C28—C29—C30	2.4 (3)
C12—C1—C5—C4	−179.27 (15)	C27—C28—C29—C30	−174.23 (17)
N1—C1—C5—C6	177.88 (15)	C28—C29—C30—C31	−2.3 (3)
C12—C1—C5—C6	−0.3 (2)	C29—C30—C31—C32	0.4 (4)
C4—C5—C6—C7	179.10 (19)	C30—C31—C32—C33	1.5 (5)
C1—C5—C6—C7	0.2 (3)	C29—C28—C33—C32	−0.6 (3)
C5—C6—C7—C8	−0.5 (3)	C27—C28—C33—C32	176.1 (2)
C6—C7—C8—C9	−178.54 (19)	C31—C32—C33—C28	−1.3 (4)
C6—C7—C8—C12	1.0 (3)	O7—C34—C35—C40	178.8 (3)
C12—C8—C9—C10	0.5 (3)	O8—C34—C35—C40	0.0 (3)
C7—C8—C9—C10	179.98 (18)	O7—C34—C35—C36	0.4 (3)
C8—C9—C10—C11	0.0 (3)	O8—C34—C35—C36	−178.4 (2)
C12—N2—C11—C10	−0.1 (3)	C40—C35—C36—C37	−0.1 (4)
Eu1—N2—C11—C10	177.34 (14)	C34—C35—C36—C37	178.3 (2)
C9—C10—C11—N2	−0.3 (3)	C35—C36—C37—C38	−0.8 (5)
C11—N2—C12—C8	0.7 (2)	C36—C37—C38—C39	1.3 (6)
Eu1—N2—C12—C8	−176.87 (11)	C37—C38—C39—C40	−0.8 (6)
C11—N2—C12—C1	−178.68 (14)	C36—C35—C40—C39	0.6 (4)
Eu1—N2—C12—C1	3.79 (17)	C34—C35—C40—C39	−177.8 (3)
C9—C8—C12—N2	−0.9 (2)	C38—C39—C40—C35	−0.1 (5)
C7—C8—C12—N2	179.62 (15)		

Symmetry codes: (i) −*x*+2, −*y*, −*z*+1.

### Hydrogen-bond geometry (Å, °)

**Table:**

Cg1 is the centroid of the C35–C40 benzene ring.

**Table:**

*D*—H···*A*	*D*—H	H···*A*	*D*···*A*	*D*—H···*A*
O8—H1O8···O1	0.86 (3)	1.82 (3)	2.660 (2)	166 (3)
C2—H2A···O6^i^	0.93	2.43	3.017 (2)	121
C9—H9A···O2^ii^	0.93	2.51	3.258 (2)	138
C11—H11A···O5	0.93	2.44	3.079 (2)	126
C25—H25A···Cg1^iii^	0.93	2.65	3.551 (3)	164

Symmetry codes: (i) −*x*+2, −*y*, −*z*+1; (ii) *x*−1, *y*, *z*; (iii) −*x*+1, −*y*, −*z*+1.
